# The role of HERV envelope protein in ovarian cancer

**DOI:** 10.3389/fcell.2025.1618542

**Published:** 2025-07-31

**Authors:** Jianhao Zhang, Dongyu Sun, Yuqing Zhan, Qing Gao, Chenxuan Bao, Huayuan Xiang, Yuxuan Shen, Qianqian Gao, Mengyu Zhang, Jianjun Wang, Lingxiang Mao

**Affiliations:** Department of Laboratory Medicine, Affiliated Kunshan Hospital of Jiangsu University, Kunshan, Jiangsu, China

**Keywords:** human endogenous retroviruses (HERVs), ovarian cancer (OC), epithelial ovarian cancer (EOC), envelope (env), herv-k

## Abstract

Human endogenous retroviruses (HERVs) are a remnant of repeated exogenous retroviral infections in human ancestors, which have been integrated into germline cells and proliferated through retrotransposition, recombination, and reinfection. Comprising approximately 8% of the human genome, HERV genes are capable of upregulating the expression of their encoded gene products in response to both endogenous and exogenous stimuli. Among HERV gene products, the envelope (env) proteins are currently extensively investigated for their pathogenic properties in cancer. Given that HERV was initially discovered in the germline cells and the ovary is an essential female reproductive organ, this review will focus on the current knowledge of the role of HERV env protein in ovarian cancer (OC). Our review systematically delineates the expression of HERV env protein across different histological subtypes of OC and highlights its pivotal roles in tumorigenesis and cancer progression. Elucidating the role of HERV env protein in OC offers novel perspectives for developing diagnostic approaches and therapeutic monitoring strategies in OC management.

## 1 Introduction

Human endogenous retroviruses (HERVs) originate from ancestral exogenous retroviral infections, where the proviral element became stably integrated into the host genome and was subsequently vertically transmitted through germline cells to progeny generations (Johnson, 2019). In the scenario of germ cell infection, the integrated retroviral element is transmitted in a Mendelian pattern and disseminated across all nucleated cells of the organism (Bannert and Kurth, 2006). HERVs became stable components of the human genome, constituting roughly 8% of our DNA (Lander et al., 2001; Morozov and Morozov, 2021). Under normal physiological conditions, HERVs remain transcriptionally silent; however, they can be aberrantly activated in various pathological states, comprising cancers (Ko et al., 2021; Costa and Vale, 2023; Cherkasova et al., 2024; Dolci et al., 2025), neurodegenerative pathologies (Gruchot et al., 2023; Kitsou et al., 2023), and autoimmune diseases (Posso-Osorio et al., 2021; Rangel et al., 2022). The envelope (env) protein, a key product of HERV activation, is a transmembrane glycoprotein encoded by the *env* gene within the HERV genome (Grandi and Tramontano, 2018; Gao et al., 2020). As shown by multiple studies, aberrant level of HERV env protein has been linked to numerous types of cancer, including ovarian cancer (OC) (Wang-Johanning et al., 2007; Salavatiha et al., 2020; Natoli et al., 2021), breast cancer (Wang-Johanning et al., 2008; Liang et al., 2024), melanoma (Büscher et al., 2005), prostate cancer (Manca et al., 2022), lung cancer (Zare et al., 2018), and colorectal cancer (Kang et al., 2023; Dolci et al., 2025).

Germ cells act as principal vectors for the vertical propagation of HERVs within the human genome, and the ovary—a vital female reproductive organ—plays a central role in oogenesis and hormonal regulation. Nearly 70% of OC has diagnostic recognition at an advanced stage, while the pathogenesis of this disease remains inadequately characterized (Lheureux et al., 2019). It remains imperative to understand the molecular pathogenesis of OC better, and elucidating the role of HERV in OC may provide novel perspectives into the pathogenic mechanisms underlying this disease. Numerous studies have demonstrated that HERV env protein is maintained at minimal levels in benign or normal ovarian tissues but exhibits significant upregulation in OC (Wang-Johanning et al., 2007; Attermann et al., 2018; Ko et al., 2021; Kitsou et al., 2023). The investigation into the function of HERV env proteins in OC holds promise for uncovering novel early-stage tumor biomarkers and advancing the development of personalized therapeutic strategies (Rycaj et al., 2015; Natoli et al., 2021).

Based on the tissue origin and pathological features, OC is mainly classified into three main categories: epithelial ovarian cancer (EOC), malignant ovarian germ cell tumor (MOGCT), and sex cord-stromal tumors (SCST) (Morgan et al., 2011; Gaona-Luviano et al., 2020). EOC constitutes the predominant histological subtype of OC (∼90%), MOGCT comprises the least OC (3–4%), and SCST is approximately 6% of OC (Chan et al., 2006; Lheureux et al., 2019; Gaona-Luviano et al., 2020). EOC is a heterogeneous disease commonly classified into five major histotypes of invasive disease: high-grade serous carcinoma (HGSOC), low-grade serous carcinoma (LGSOC), mucinous carcinoma (MOC), endometrioid carcinoma (ENOC), and clear cell carcinoma (CCOC) (Phelan et al., 2017; Sambasivan, 2022). MOGCT is predominantly categorized into five types: dysgerminoma, yolk sac tumor, immature teratoma, embryonal carcinoma, and mixed germ cell tumors (Kraggerud et al., 2013). With a low prevalence of SCST and the three histologic subtypes of MOGCT—immature teratoma, embryonal carcinoma, and mixed germ cell tumors, the function of HERV env protein in them has been less investigated. Therefore, this review will mainly address the role of HERV env protein in EOC and two main types of MOGCT—dysgerminoma and yolk sac tumor (Table 1).

**TABLE 1:** Comprehensive overview of HERV env in OC.

Tumor type	HERV type	Gene	Detection	Sample	Description	References
Epithelial ovarian cancer (EOC)	HEMO	*env*	Protein	Tissue	The transcriptional activity of HEMO is upregulated in CCOC tissues	Heidmann et al. (2017)
	HERV-K	*env*	Protein	Tissue	HERV-K env expression is inversely correlated with OC malignancy potential and histologic grade	Wang-Johanning et al. (2007)
	HERV-R	*env*	Protein	Tissue	HERV-R env protein levels were markedly higher in stage I in contrast to stages II-IV	Jeon et al. (2020)
	HERV-W	Promoter regions	Protein	Tissue	Hypomethylation of HERV-W promoter CpG sites drives env transcriptional activation	Menendez et al. (2004)
	HERV-E, HERV-K, and ERV3	*env*	mRNA	Tissue	Higher levels of HERV-E, HERV-K, and ERV3 relative to those in normal ovarian epithelial tissues	Wang-Johanning et al. (2007), Kang et al. (2014)
	HERV-K	*env*	Transcript	Tissue	Both HERV-K env spliced Rec and Np9 transcripts were detected in ovarian serous carcinoma tissues	Wang-Johanning et al. (2007)
	HERV-K	*env*	Protein	Cell	High expression levels of HERV-K env protein were observed on the cell surface and in the cytoplasm of EOC cells	Wang-Johanning et al. (2007)
	HERV-K	*env*	Transcript	Cell	Both HERV-K env spliced Rec and Np9 transcripts were detected in EOC cells (DOV13 and SKOV3)	Wang-Johanning et al. (2007)
	HERV-E, HERV-K, and ERV3	*env*	Antibody	Plasma from patients	Anti-HERV-K env titers were significantly higher than anti-HERV-E and anti-ERV3	Wang-Johanning et al. (2007), Rycaj et al. (2015)
	HERV-K	*env*	Protein	Ascites-derived cells from patients	Both primary and metastatic ascites-derived cells and ascites samples exhibited elevated HERV-K env protein on their surface	Rycaj et al. (2015)
Dysgerminoma	HERV-K	*gag* and *env*	RNA	Tissue	Dysgerminoma tissues shared HERV-K expression of gag and env RNA	Herbst et al. (1996)
	HERV-K	*gag, pol, a*nd *env*	RNA transcript	Tissue	High levels of the corresponding RNA transcripts were observed through non-overlapping probes	Herbst et al. (1996), Herbst et al. (1999)
Yolk sac tumor	HERV-K	*gag* and *env*	RNA	Tissue	HERV-K gag and env RNA are detected in yolk sac tumor tissues	Herbst et al. (1996)
	HERV-K	*env*	mRNA	Cell	Yolk sac tumor cells show an intermediate level of HERV-K env mRNA	Mueller et al. (2018)
	HERV-K	*gag* and *env*	Antibody	Plasma from patients	Serum from yolk sac tumor patients exhibited significantly higher anti-HERV-K gag and env antibodies	Kleiman et al. (2004), Curty et al. (2020)

## 2 The structure, classification, and activation of HERV

**FIGURE 1:**
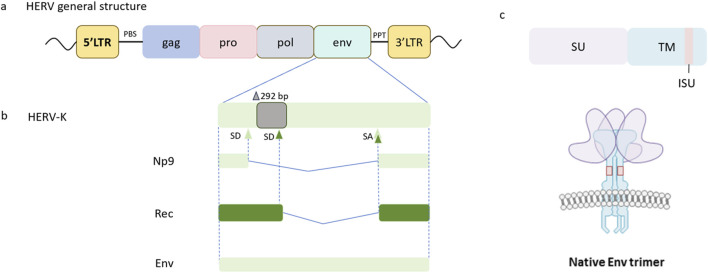
**(a)** Genomic structure of HERV sequence. A complete sequence of HERVs comprises *gag, pro, pol*, and *env* regions packed between two LTRs. The primer binding site (PBS) flanks the 5′LTR and precedes the *gag* gene, and the polypurine tract (PPT) is positioned downstream of the *env* gene adjacent to the 3′LTR. Adapted from this article (Küry et al., 2018). **(b)** The HERV-K *env* gene exhibits alternative splicing sites, resulting in the presence of structurally full-length transcripts and two types of accessory variants termed Rec and Np9, which subdivide HERV-K sequences into two subtypes. Type I HERV-K elements display a characteristic 292-bp deletion in the *env* region, leading to the utilization of an upstream splice donor site (SD) and subsequent production of a shorter protein designated Np9, whereas Type II HERV-K sequences retain this segment and employ a downstream SD to encode a longer protein named Rec. Adapted from this document (Grandi and Tramontano, 2018) **(c)** The HERV-K env protein consists of surface glycoprotein (SU) and transmembrane (TM) subunits and the TM subunit contains the immunosuppressive domain (ISD). The HERV-K surface env protein assembles into a trimeric structure, consisting of heterodimers formed by SU and TM subunits. The SU subunit is responsible for receptor binding, and the TM subunit plays a role in immunomodulation. Adapted from this document (Nadeau et al., 2015). SA, Splice acceptor site.

HERVs are a distinct class of retrotransposons embedded in the human genome and are pivotal agents of genome evolution (Hughes and Coffin, 2002; Luqman-Fatah et al., 2024). The canonical genomic organization of HERVs comprises four core genes—*gag, pro, pol*, and *env*—encoding structural and enzymatic proteins, flanked by two LTRs (Xue et al., 2020; Liu et al., 2023). LTR harbors core promoter and enhancer elements that orchestrate transcriptional regulation of both HERV-derived sequences and adjacent host genes through epigenetic modifications and transcription factors recruitment (Jern et al., 2005; Ito et al., 2017). The specific functions of the HERV components are shown in (Figure 1a). The primer binding site (PBS) is positioned between 5′LTR and *gag*, and the polypurine tract (PPT) is located between *env* and 3′LTR. The *gag* gene encodes the structural components including capsid, nucleocapsid, and matrix protein. The *pro* gene encodes a viral protease called dUTPse. The *pol* gene generates viral enzymes including reverse transcriptase (RT), Ribonuclease H (RNase H), and integrase (Bannert and Kurth, 2006; Küry et al., 2018; Duarte et al., 2024). The *env* gene encodes env protein consisting of a 55 kDa surface glycoprotein (SU), which determines the specificity of host cell receptor recognition, and a 39 kDa transmembrane (TM) subunit, which is vital in anchoring the viral receptor to the host cell membrane and facilitating the fusion process between viral particles and the host cell, ensuring successful viral entry. TM subunit contains the immunosuppressive domain (ISD) involved in host immune regulation (Löwer et al., 1996; Nadeau et al., 2015; Pisano et al., 2019) (Figure 1c). The HERV-K *env* gene is capable of producing not only the env protein but also two proteins of distinct lengths, namely Np9 and Rec, depending on the presence or absence of a 292-bp deletion, proposed to have oncogenic properties (Chen et al., 2013; Hanke et al., 2013; Soleimani-Jelodar et al., 2024) (Figure 1b). Rec is a 14 kDa accessory protein that functionally serves as the Rev and Rex proteins for HIV and HTLV respectively, and Np9 is a 9 kDa protein identical to its first 14 amino acids with HERV-K Rec (Hanke et al., 2013; Chan et al., 2019; Fan and Qin, 2024).

Due to the lack of proper nomenclature and the continuous increase in the knowledge of HERV, the classification of HERVs has been incomplete for a long time and is constantly being revised. The conventional nomenclature of HERV subtypes refers to the first-letter amino acid code of the tRNA of the primary binding site during the reverse transcription process (e.g., HERV-K for lysine, HERV-H for histidine, and HERV-W for tryptophan) (Tristem, 2000; Vargiu et al., 2016). Nevertheless, this naming approach has notable limitations, particularly when distinct HERV families utilize the same tRNA species (Bannert and Kurth, 2004). Thus, alternative nomenclature strategies have been adopted in some cases, incorporating neighboring gene names (e.g., HERV-ADP), and clone numbers (e.g., HERV-S71) (Salavatiha et al., 2020). The major members of HERVs are divided into three principal categories. Class I HERVs are classified within the *Gammaretrovirus* and *Epsilonretrovirus* genera, exhibiting a multi-layered evolutionary architecture that encompasses distinct supergroups and canonical subgroups. For detailed phylogenetic reconstructions and taxonomic criteria, readers can consult this article (Vargiu et al., 2016). Class II belongs to *Betaretrovirus*, encompassing the HERV-K family, which is further subdivided into ten distinct subgroups (HML-1 to HML-10). Class III is affiliated with *Spumaretrovirus*, which comprises HERV-L and HERV-S (Vargiu et al., 2016; Dopkins and Nixon, 2024). HERV-K(HML-2) stands out as the most extensively well-characterized member, with substantial evidence implicating its role in oncogenic processes (Rivas et al., 2022; Costa and Vale, 2023).

In most human tissues under physiological conditions, HERV expression remains epigenetically silenced and is generally undetectable at baseline levels (She et al., 2022; Dopkins and Nixon, 2024). However, HERV genomic elements can be transcriptionally activated in response to various external stimuli, including exogenous chemicals (Johnston et al., 2001), physical conditions (Reiche et al., 2010; Lee et al., 2012), and exogenous viral infections (Contreras-Galindo et al., 2007). Mechanistic studies show that phorbol-12-myristate-13-acetate (PMA) induces three to nine -fold upregulation of HERV-K transcripts in primary macrophages and monocytes (Johnston et al., 2001). It has been demonstrated that physical conditions including X-rays and UVB irradiation function as facilitators of HERV transcriptional activity (Reiche et al., 2010; Lee et al., 2012). Of particular clinical relevance, HIV-1 infection has been observed to amplify HERV-K expression in CD4 (+) T lymphocytes isolated from peripheral blood mononuclear cells (PBMCs) (Contreras-Galindo et al., 2007).

## 3 The role of HERV env protein in EOC

EOC is the predominant histological subtype of OC, arising from malignant transformation of ovarian surface epithelial cells or fallopian tube epithelium (Siegel et al., 2022). This aggressive malignancy is characterized by frequent late-stage diagnoses and accounts for the majority of gynecological cancer mortality (Lheureux et al., 2019). Notably, elevated levels of HERV env protein in EOC, as opposed to benign ovarian tumors and normal ovarian epithelial tissue, have been reported in multiple studies (Rycaj et al., 2015; Kitsou et al., 2023; Kim et al., 2024).

Emerging evidence has characterized a novel HERV env protein, designated as HEMO [human endogenous MER34 (medium-reiteration-frequency-family-34) ORF], which illustrates marked upregulation of transcriptional activity in CCOC tissues compared to normal ovarian epithelium and other histologic subtypes of EOC (Heidmann et al., 2017). The elevation of HERV-K and HERV-R env proteins has also been revealed in EOC cells and tissues (Jeon et al., 2020; Ko et al., 2021; Kim et al., 2024). To investigate the subcellular distribution of HERV-K env protein, flow cytometry analysis was conducted to elucidate high expression levels on the cell surface and in the cytoplasm of EOC cells. Immunohistochemical analysis of multi-tissue microarrays further quantified the frequency of HERV-K env protein expression across different EOC tissue samples. During the progression of EOC, levels of HERV-K env were notably upregulated in tumors with low malignant potential and low-grade, including low malignant potential serous tumors (LMP serous tumors), LGSOC, and low-grade endometrioid carcinoma (LGENOC), relative to their high malignant potential and high-grade counterparts (Wang-Johanning et al., 2007). Consistent with the expression pattern of HERV-K env protein, HERV-R env protein levels were markedly higher in stage I tissues in contrast to those in the advanced stage (stages II-IV) (Jeon et al., 2020). Furthermore, the histologic analysis indicated that transitional cell carcinomas and endometrioid adenocarcinomas (a subtype of ENOC) exhibited marginally higher HERV-K env expression levels than other histologic subtypes of EOC (Wang-Johanning et al., 2007). The upregulation of HERV-R env protein in serous papillary adenocarcinoma (a subtype of HGSOC) was markedly compared to other histologic types of EOC (Jeon et al., 2020). In addition to the observations in EOC cell lines, indirect immunofluorescence microscopy analysis confirmed the presence of HERV-K env protein on the surface of both primary and metastatic ascites-derived cells obtained from EOC patients. Notably, comparative analysis showed that ascite samples consistently exhibited elevated levels of env protein expression when relative to either primary tumor tissues or benign lesions (Rycaj et al., 2015). It is intriguing that ascites typically manifests in the later stages of EOC, which is distinct from previous findings indicating that env proteins of HERV-K and HERV-R are upregulated during the early stages of EOC. The underlying mechanisms responsible for this apparent discrepancy warrant further investigation through comprehensive molecular and clinical studies.

Congruent with the elevated expression of HERV env proteins in EOC cells and tissues described above, RT-qPCR analysis of HERV env mRNA in EOC tissues indicated higher levels of HERV-E, HERV-K, and endogenous retroviruses type 3 (ERV3) in contrast to those in normal ovarian epithelial tissues (Wang-Johanning et al., 2007; Kang et al., 2014). Spliced transcripts of HERV-K env-derived Rec and Np9 were identified in EOC cell lines DOV13 and SKOV3, as well as ovarian serous carcinoma tissues (Wang-Johanning et al., 2007). Several studies uncovered that HERV env mRNA was not notably elevated in EOC cells compared to benign ovarian epithelial cells. Low levels of HERV-E and HERV-K env mRNA in matched uninvolved normal ovarian tissues were detected. Sequence analysis revealed several stop codons in uninvolved ovarian tissues, indicating that no full-length env protein could be translated, by contrast, stop codons were not found in HERV env mRNAs extracted from EOC tissue. This suggests that despite low levels of HERV-E and HERV-K env mRNA expression in normal ovarian tissues, HERV env mRNA could not be translated in normal ovarian tissues while it could be translated in EOC (Seifarth et al., 2005; Wang-Johanning et al., 2007). The presence of anti-HERV env protein antibodies provides indirect evidence of HERV env protein in EOC. Anti-HERV antibodies, including anti-HERV-K env protein, anti-HERV-E env protein, and anti-ERV3 env protein were detected in the sera of EOC patients but not in normal female controls. Besides, anti-HERV-K env antibody titers were markedly higher than the other two types of antibodies in the same test sera (Seifarth et al., 2005; Rycaj et al., 2015).

To explore the impact of the HERV-K env on the genesis of EOC cell lines, the HERV-K *env* gene was knocked out in EOC cell lines SKOV3 and OVCAR3 through the CRISPR-Cas9 system, which indicated dramatic attenuation of tumor cell proliferation, migration, and invasion (Ko et al., 2022; Ko et al., 2024). Correlative analysis between HERV-K env and cancer stem cell markers in EOC cells revealed notable suppression of stemness-associated markers following CRISPR/Cas9-mediated HERV-K *env* gene knockout, establishing a critical regulatory role of the retroviral element in maintaining malignant stem-like properties in EOC (Kim et al., 2024). Furthermore, HERV-W represents a family of LTR retrotransposons, comprising roughly 140 full-length or truncated elements randomly distributed throughout the human genome (Kim and Lee, 2001). A comparative analysis of the methylation status of CpG dinucleotides within the promoter regions of these elements was conducted between EOC tissues and normal ovarian epithelial tissues, utilizing a restriction-enzyme based assay. The results disclosed hypomethylation at CpG dinucleotides in the HERV-W promoter regions of EOC tissues, which was found to critically regulate the transcriptional activation and subsequent expression of the *env* gene (Menendez et al., 2004).

A potentially useful source of tumor-associated antigens (TAA) for therapeutic vaccination targeting may be found in the HERV-K env protein. The development of a cancer vaccine for EOC based on HERV-K env protein functioned as TAA revealed that HERV-K env-specific cytotoxic T lymphocytes (CTLs) can elicit robust cytotoxicity towards autologous EOC cells. Compared to benign ovarian disease cells that hardly exhibit expression of HERV-K env, HERV-K env-specific CTLs demonstrated preferential cytotoxicity against autologous EOC cells with HERV-K env expression. Furthermore, PBMCs isolated from EOC patients were found to harbor HERV-K env-specific CTLs, which were capable of mounting cytolytic responses against HERV-K env-positive EOC cells upon *in vitro* reactivation (Rycaj et al., 2015). A thorough exploration of HERV in EOC was conducted by combining data from The Cancer Genome Atlas (TCGA) with an independent dataset obtained from Hammersmith Hospital. This study uncovered that a distinct HERV expression signature not only serves as a prognostic indicator for HGSOC but also indicates a profound correlation with enhanced effector T cell infiltration within the tumor microenvironment. Furthermore, experiments *in vitro* suggested that upregulated baseline HERV expression may contribute to increased tumor immunogenicity and potentially influence therapeutic responsiveness to DNA methyltransferase inhibitors (DNMTi). This implies that manipulation of the expression of HERV by DNMTi resulted in improved EOC cell killing by cytotoxic immune cells, and the serum HERV expression scores of EOC patients can predict the level of immune infiltration is anticipated to be one of the most crucial markers for both diagnosis and disease progression tracking (Natoli et al., 2021).

## 4 The role of HERV in two main classes of MOGCT

### 4.1 Dysgerminoma

Dysgerminoma is one of the most common MOGCTs, occurring mainly in women under 30 years of age, with similarities in pathologic features to the classic testicular spermatogonia seminoma (Mitranovici et al., 2022). Patients with dysgerminoma are sensitive to chemotherapy and have a good prognosis with an overall survival rate of more than 90% (Shaaban et al., 2014; Friedrich et al., 2025), therefore, few studies have been conducted on the pathogenesis of this tumor. Utilizing non-overlapping, isotopically labeled RNA probes specific for HERV-K gag and env, *in situ* hybridization revealed that dysgerminoma tissues shared HERV-K expression of gag and env RNA (Herbst et al., 1996). High levels of the corresponding RNA transcripts were observed through non-overlapping probes targeting the *gag, pol,* and *env* genes of the prototypical proviral sequence HERV-K (Herbst et al., 1996; Herbst et al., 1999). At present, no proof is evident in the literature that HERV proteins are detected in dysgerminoma and the underlying function of HERV env protein in dysgerminoma can be further investigated by novel techniques, such as gene microarray, single-cell sequencing, and mass spectrometry.

### 4.2 Yolk sac tumor

Yolk sac tumor, representing the second most prevalent MOGCTs, are characterized by their complex histopathological composition, including endoderm-like differentiated extra-embryonic tissues, immature embryonic endodermal derivatives, and mesenchymal components (Ramalingam, 2023). The summary of situ hybridization results indicates that HERV-K gag and env RNA are detected in yolk sac tumor tissues, consistent with dysgerminoma (Herbst et al., 1996). Mueller et al. (2018) uncovered that yolk sac tumor cells expressed pluripotency markers LIN28A and showed an intermediate level of HERV-K env mRNA higher than somatic differentiated germ cell tumor cells. Moreover, HERV-K gag protein can be detected by immunoblotting in tumor biopsies (Herbst et al., 1996). Significantly higher levels of anti-HERV-K gag and anti-HERV-K env can be observed in serum samples taken from patients with yolk sac tumor (Kleiman et al., 2004; Curty et al., 2020). Nevertheless, further experimental validation is required to precisely quantify the upregulated expression of HERV-derived proteins in yolk sac tumor specimens and elucidate their potential role in disease pathogenesis or progression.

## 5 Regulation of HERV env expression-HERV LTR

HERV LTR precisely regulates HERV env expression through its promoters, enhancers, transcription factor binding sites, and epigenetic regulatory mechanisms (Dopkins and Nixon, 2024). Transcriptional activation of the HERV LTR drives elevated expression of the *env* gene, concomitant with overexpression of encoded oncoproteins (Büscher et al., 2005). For the transcription of human retroviral genes, LTRs enlist transcription factors from the infected cell and have the ability to increase host cell gene transcription, which can result in unchecked tumor cell proliferation (María et al., 2016). In addition to initial involvement in retroviral integration in the host genome, LTRs can also serve as alternative promoters and enhancers, leading to dysregulated gene expression that could aid in tumorigenesis.

P53 may modulate the transcription of the HERV LTR through direct binding, with its regulatory effects—either activation or repression, depending on the cellular environment and the sequence context of the LTRs. Chang et al. (2007) isolated the LTR of RTVL-Ia (a prototypical member of the HERV type I family) and concluded that HERV-I LTR may become active upon p53 mutation. Additionally, Chromatin immunoprecipitation (ChIP) followed by qPCR was performed to determine the contact between LTR5Hs (most active LTR of HERV-K fragments) and p53 protein, and the results showed that the two p53 binding sites in LTR5Hs play a critical role in regulating the transcriptional activity of LTR5Hs (Wang et al., 2007; Liu et al., 2022). It would be intriguing to ascertain how mutant p53-mediated activation of LTRs may promote the incorporation of HERV env protein into oncogenic signaling cascades, thereby triggering retroelement mobilization and inducing epigenetic disruption of tumor suppressor genes. This potential mechanism could provide a novel link between HERV *env* gene activation and the oncogenic transformation of cells.

## 6 Conclusion and perspectives

Although there are numerous subgroups of HERV-K that have been extensively studied in tumors, international uniform standards of HERV classification have not yet been determined. This makes the advancement of HERV-related research relatively challenging on a global scale. Besides, most studies report significantly upregulated expression of HERV-K env in OC, but few studies indicate no significant difference in HERV-K env expression between OC tissues and normal ovarian tissues (Ko et al., 2021). This discrepancy may require further investigation with expanded sample sizes for validation. Overall, upregulation of env from various HERV subtypes has been observed in OC, while their clinical associations and potential pro-tumorigenic roles during cancer progression require further exploration.

The pathognomonic overexpression of HERV env protein in cancer cells has emerged as a promising source of biomarkers for cancer diagnosis and therapeutic monitoring (Alcazer et al., 2020). For example, HERV-K102 env in circulating blood as an immunomodulatory biomarker capable of evaluating both immunosuppressive status and disease staging in cancer patients with pancreatic ductal adenocarcinoma (PDAC), hepatocellular carcinoma (HCC), and non-small cell lung cancer (NSCLC) (Gong and Xu, 2025). Future research should focus on conducting large-scale cohort studies to validate the diagnostic potential of HERV env protein as a molecular marker for OC. Furthermore, the tumor-specific expression of HERV env protein and the subsequent induction of anti-HERV immune responses present novel opportunities for cancer immunotherapy development. Recent evidence demonstrates the promising efficacy of therapeutic cancer vaccines targeting endogenous retroviral protein ERVMER34-1 in combination with immune checkpoint inhibitors against select malignancies (Maldonado et al., 2025). Although targeting HERV env proteins represents a promising tumor-specific strategy, current evidence supporting their immunotherapeutic value is largely derived from murine models (Zhou et al., 2015; Maldonado et al., 2025). Clinical therapies targeting HERV-derived antigens are now being explored in humans, necessitating comprehensive safety evaluations to mitigate potential adverse effects and ensure efficacy.
